# Lack of the Nlrp3 Inflammasome Improves Mice Recovery Following Traumatic Brain Injury

**DOI:** 10.3389/fphar.2017.00459

**Published:** 2017-07-14

**Authors:** Natasha Irrera, Gabriele Pizzino, Margherita Calò, Giovanni Pallio, Federica Mannino, Fausto Famà, Vincenzo Arcoraci, Vincenzo Fodale, Antonio David, Cosentino Francesca, Letteria Minutoli, Emanuela Mazzon, Placido Bramanti, Francesco Squadrito, Domenica Altavilla, Alessandra Bitto

**Affiliations:** ^1^Department of Clinical and Experimental Medicine, AOU Policlinico G. Martino, University of Messina Messina, Italy; ^2^Department of Veterinary Sciences, University of Messina Messina, Italy; ^3^Department of Human Pathology, AOU Policlinico G. Martino, University of Messina Messina, Italy; ^4^IRCCS Centro Neurolesi “Bonino-Pulejo” Messina, Italy; ^5^Department of Biomedical and Dental Sciences and Morphological and Functional Sciences, AOU Policlinico G. Martino, University of Messina Messina, Italy

**Keywords:** NLRP3 inflammasome, traumatic brain injury, inflammation, cytokines, apoptosis

## Abstract

Treatment for traumatic brain injury (TBI) remains elusive despite compelling evidence from animal models for a variety of therapeutic targets. The activation of the NLRP3 (Nucleotide-binding oligomerization domain-like receptor family pyrin domain-containing 3) inflammasome has been proposed as key point in the brain damage associated with TBI. NLRP3 was tested as potential target for reducing neuronal loss and promoting functional recovery in a mouse model of TBI. Male NLRP3^-/-^ (*n* = 20) and wild type (*n* = 27) mice were used. A closed TBI model was performed and inflammatory and apoptotic markers were evaluated. A group of WT mice also received BAY 11-7082, a NLRP3 inhibitor, to further evaluate the role of this pathway. At 24 h following TBI NLRP3^-/-^ animals demonstrated a preserved cognitive function as compared to WT mice, additionally brain damage was less severe and the inflammatory mediators were reduced in brain lysates. The administration of BAY 11-7082 in WT animals subjected to TBI produced overlapping results. At day 7 histology revealed a more conserved brain structure with reduced damage in TBI NLRP3^-/-^ animals compared to WT. Our data indicate that the NLRP3 pathway might be exploited as molecular target for the short-term sequelae of TBI.

## Introduction

Traumatic brain injury (TBI) and its sequelae are a common cause of death and disability in young adults all over the world (Maas et al., 2008). TBI is a pathologic condition characterized not only by a direct damage in brain but also by harmful secondary pathologic processes such as inflammation, oxidative-nitrosative stress, necrosis and apoptosis (Wang et al., 2007; Brouns and De Deyn, 2009). All these events may be responsible for physical disabilities as well as long-term cognitive, behavioral, psychological, and social deficits that influence lifestyle in TBI patients (Kurth et al., 1994).

The mechanisms involved in the “secondary phase” of TBI are not completely understood but several studies have demonstrated that inflammatory response plays a key role (Hang et al., 2006; Zhao et al., 2011).

Glial cells, in particular microglia and astrocytes, are the main source of inflammatory molecules. Physiologically, these cells are involved in neuronal activity and cerebral homeostasis. In stress conditions, glial cells produce neurotoxic molecules, including reactive oxygen species (ROS) and proinflammatory cytokines such as Tumor Necrosis Factor alpha (TNF-α) and interleukins (e.g., IL-6 and IL-1β) (Reale et al., 2009; Yan et al., 2009).

Reactive oxygen species accelerate neurodegeneration process, in particular at axonal level, and cause lipid peroxidation of cell membranes. This membrane impairment is responsible for cellular necrosis and for the consequent release of mediators of inflammation from glia (Povlishock and Kontos, 1992; Bell, 2013). Additionally, increased ROS levels modify intracellular calcium concentrations; this ionic imbalance may also lead to the discharge of ATP that may also act as a stressor for neurons, initiating innate immune activation and contribute to cellular death following traumatic injuries (Russo and McGavern, 2015).

ATP acts by binding purinergic receptors such as purine P2X7 receptors (Ralevic and Burnstock, 1998), thus contributing an inflammatory cascade. These receptors are particularly triggered during TBI (Kimbler et al., 2012) and their activation has been proposed to be the upstream signal of NLRP3 inflammasome assembly (Ferrari et al., 2006). NLRP3 inflammasome consists of NLRP3, apoptotic speck-containing protein (ASC) adaptor, and procaspase-1; when NLRP3 is activated, procaspase-1 is cleaved into caspase-1, which mediates the secretion of the pro-inflammatory cytokines IL-1β and IL-18 (Martinon, 2010). A switch between pyroptosis and apoptosis may be possible and it is mediated by ASC: intracellular pathogens and their products lead to activation of the ASC-dependent inflammasome. Strong inflammasome activation leads to caspase 1 recruitment and activation, and associated pyroptosis, IL-1β and IL-18 activation, and inflammation. Weak inflammasome activation promotes ASC-mediated caspase 8 recruitment and activation of the apoptotic pathway. Depending on the signaling (caspase-3 or BAX), the mitochondrial signaling pathway may or may not be engaged (Grabinger et al., 2013).

It has been demonstrated that NLRP3 inflammasome is involved in several neurological disorders, in particular those associated with inflammation, such as Alzheimer’s and Huntington’s diseases (Halle et al., 2008; Jha et al., 2010).

Some studies have indicated that NLRP3 may be found both in microglia and in astrocytes (Halle et al., 2008; Jha et al., 2010); however, it has been demonstrated that neural cells express NLRP1 inflammasome, but not the NLRP3 inflammasome (de Rivero Vaccari et al., 2008; Silverman et al., 2009), whereas a recent study showed that NLRP3 is expressed also in neurons of TBI rats (Liu et al., 2013). An *in vitro* study has demonstrated that “knocking down” and “knocking out” the expression of ASC and NLRP3 reduced the production of the pro-inflammatory cytokine IL-1β (Craven et al., 2009), thus confirming that NLRP3 might be targetable to modulate inflammatory processes.

In light of all these observations, inflammation represents one of the main problems in TBI and its exacerbation worsens the prognosis of the disease. The present study evaluated whether the lack of the NLRP3 inflammasome might ameliorate the course of TBI, reducing inflammation and modulating the apoptotic machinery. For this reason, mice lacking the gene encoding for NLRP3 (NLRP3^-/-^) and their normal littermates were used to appreciate the role of the NLRP3 inflammasome pathway in a model of TBI.

## Materials and Methods

### Animals and Group Allocation

Male NLRP3 knock-out (KO) mice (NLRP3^*Tm*1*Bhk/g*^) (*n* = 20) and their normal littermates (C57Bl6/J *n* = 27) (Charles River, Calco, Italy) were used in this study. Animals were housed in plastic cages, maintained under controlled environmental conditions (12 h light–dark cycle, 24°C), and provided with standard food and water *ad libitum* in the Animal Facility of the Department of Clinical and Experimental Medicine of the University of Messina.

All experiments were carried out according to the standards for care and use of animals as stated in the Directive 2010/63/EU, and the ARRIVE guidelines (Kilkenny et al., 2010). The procedures were evaluated and approved by the Ethic Committee of the University of Messina (#08/15).

Animals were randomly assigned to the following groups: Sham WT (*n* = 6), Sham NLRP3^-/-^ (*n* = 6), WT-TBI (*n* = 14), WT-TBI + BAY 11-7082 (20 mg/kg/i.p.; *n* = 7), NLRP3^-/-^ -TBI (*n* = 14).

### Experimental TBI

Both NLRP3^-/-^ and WT animals were anesthetized by intraperitoneal injection of ketamine/xylazine (80 and 10 mg/kg, respectively) and subjected to an impact-acceleration model of diffuse TBI, based on the model described by Marmarou et al. (Marmarou et al., 1994), with some modifications. Briefly, an incision was made along the sagittal midline to expose the skull and a 3 mm thick steel disk was placed on between λ and bregma sutures. Animals were arranged on a 10-cm foam bed to soften the impact. Briefly, a 8 g weight with a 5 mm diameter and 5.5 cm length was dropped from a distance of 1.27 m, to produce TBI. After TBI, the steel disk was removed and the skin immediately sutured; a 2% lidocaine jelly was spread on to the impact site to reduce pain. Animals in WT-TBI and in NLRP3^-/-^-TBI group were randomly killed at 24 h (*n* = 7 from each group) or 7 days (*n* = 7 from each group) following procedures. The WT-TBI + BAY 11-7082 group received the NLRP3 inflammasome inhibitor BAY 11-7082 (Sigma–Aldrich S.r.l., Milan, Italy) 10 min after TBI procedures and were killed at 24 h or 7 days for histological and biochemical assessments.

### Behavioral Test: Novel Object Recognition

At 24 h following TBI, both NLRP3^-/-^ and WT mice were subjected to an object recognition task to evaluate recognition memory (Antunes and Biala, 2012). Before TBI, in the “acquisition phase”, animals were individually put into an open field box (59 × 59 × 20) cm containing two identical objects (A and B) located in a symmetric position for 5 minutes to get acquainted with the new space. The two objects were adequately heavy and high so that mice could not move or climb over them. Twenty-four hours after TBI, one object, A or B, was substituted for a novel one (C) and exploratory behavior was again evaluated for 5 min. For each evaluation, both box and all objects were carefully cleaned with 70% ethanol to remove every odor recognition. Exploration of an object was evaluated whenever the mice rearing on or sniffing an object at a distance of less than 2 cm and/or touching it with the nose. It was considered a successful recognition if the animal preferred the exploration of the novel object. Discrimination of visual novelty was assessed by a preference index (Dix and Aggleton, 1999), calculated as follows: (time near the new – time near the old object)/(time near the new + time near the old object).

### ELISA Evaluation for IL-1b and Caspase-1

Brains were collected 24 h following TBI and 100 mg tissue was rinsed with 1X PBS, homogenized in 1 mL of 1X PBS and stored overnight at -20°C. After two freeze-thaw cycles were performed to break the cell membranes, the homogenates were centrifuged for 5 min at 5000 × *g* at 4°C. The supernatant was removed and assayed immediately to determine IL-1b and Caspase-1, using mouse-specific commercially available ELISA kits (Cusabio, College Park, MD, United States). Samples were run in duplicate, and the absorbance was read at 450 nm; the results were compared to the standard curves and expressed in pg/mL.

### Western Blot Analysis

Brains were collected 24 h or 7 days following TBI. Hippocampus was obtained from brains and samples were homogenized in 400 μl of RIPA buffer with proteases inhibitors [25 mM Tris/HCl (pH 7.4), 1.0 mM EGTA, 1.0 mM EDTA, 0.5 mM phenyl methylsulfonyl fluoride, aprotinin, leupeptin, pepstatin A (10 μg/mL each) and NP-40 (10 μl/mL)]. Homogenate samples were centrifuged and the supernatants were collected and used for protein determination using the Bio-Rad protein assay kit (Bio-Rad, Richmond, CA, United States).

Protein samples were denatured in reducing buffer [62 mm Tris (pH 6.8), 10% glycerol, 2% sodium dodecyl sulfate, 5% β-mercaptoethanol, and 0.003% bromophenol blue] and separated by electrophoresis on an sodium dodecyl sulfate polyacrylamide gel (12%). The separated proteins were transferred onto a PVDF membrane using a transfer buffer [39 mm glycine, 48 mm Tris (pH 8.3), and 20% methanol] at 200 mA for 1 hour. The membranes were stained with Ponceau’s (0.005% in 1% acetic acid) to evaluate equal amounts of protein and were blocked with 5% non-fat dry milk in Tris-buffered saline (TBS)-0.1%, washed and incubated with a primary antibody for EGR-1 (1:500; Abcam, Cambridge, United Kingdom) as marker of memory function; BAX, BCL-2, β-actin (1:500; Cell Signaling, Beverly, MA, United States), BCL-XL (1:500, Milpitas, CA, United States). The membranes were then incubated with a secondary antibody peroxidase-conjugated for 1 h at RT and were analyzed by chemiluminescence (KPL, Gaithersburg, United States). Protein signals were quantified by scanning densitometry using a bio-image analysis system (C-DiGit Blot Scanner, with Image Studio software; Lincoln, NE, United States) and the results were expressed as relative integrated intensity compared to controls. β-actin was used to confirm equal protein loading and blotting.

### Histology

Brains were fixed in 10% buffered formalin, embedded in paraffin, cut in coronal sections (5 μm) and stained with hematoxylin and eosin to observe morphologic changes.

Injured neurons were counted and a six-point scale score was applied to evaluate the histopathologic changes of the gray matter (Kawai and Akira, 2007): 0, no lesion observed; 1, gray matter contained one to five eosinophilic neurons; 2, gray matter contained five to 10 eosinophilic neurons; 3, gray matter contained more than 10 eosinophilic neurons; 4, small infarction (less than one third of the gray matter area); 5, moderate infarction (one third to one half of the gray matter area); 6, large infarction (more than half of the gray matter area). An average of the scores for each group was recorded to give a cumulative score. At least three slides for each animal were examined by a blinded pathologist.

### Statistical Analysis

All data are expressed as means ± SD. Comparisons between different treatments were analyzed by one-way or two-way ANOVA for non-parametric variables with Tukey’s post-test for intergroup comparisons. Mann–Withney *U* test was used to analyze the histological scores and preference index. The possibility of error was set at *p* < 0.05 and it was considered statistically significant. All analyses were performed using Stata/IC 12.0 (StataCorp LP, College Station, TX, United States). Graphs were drawn using GraphPad Prism (version 5.0 for Windows).

## Results

### Behavioral Test and EGR1 Expression

**FIGURE 1:**
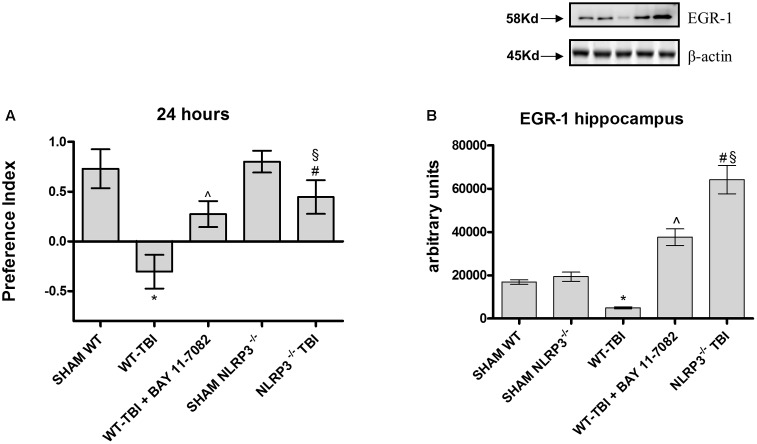
The graph **(A)** represents the cumulative score obtained by the animals in the behavioral test 24 h after TBI. Representative western blot **(B)** of EGR-1 in the hippocampus of mice evaluated at 24 h. ^∗^*p* < 0.05 vs Sham WT; ˆ*p* < 0.05 vs WT-TBI; ^#^*p* < 0.05 vs Sham NLRP3^-/-^; ^§^ *p* < 0.05 vs WT-TBI. Each bar represents the mean and SD of seven animals.

The Novel Object Recognition was performed to study the differences in cognitive performance between WT and NLRP3^-/-^ mice. WT-TBI mice had reduced memory ability at 24 h (**Figure 1A**, *p* < 0.0001), while WT-TBI+BAY 11-7082 and NLRP3^-/-^ mice showed better performance results (**Figure 1A**, *p* < 0.005).

To confirm if this data could be related to a memory preservation, a Western blot analysis was carried out to evaluate EGR1 expression in hippocampus samples at 24 hours. TBI-WT mice had significantly reduced EGR-1 expression as compared to Sham WT and Sham KO mice (*p* < 0.0001). BAY 11-7082 administration significantly improved EGR-1 in hippocampal tissue as compared to either WT-TBI and Sham WT animals (*p* < 0.0001). NLRP3^-/-^ mice had increased EGR-1 levels following TBI as compared to Sham KO (*p* < 0.0005) and TBI-WT (*p* < 0.01) animals, thus suggesting an improvement in the initiation of mechanisms underlying consolidation of memory (**Figure 1B**, *F* = 297.2).

### NLRP3 Absence Reduces Brain Inflammation

**FIGURE 2:**
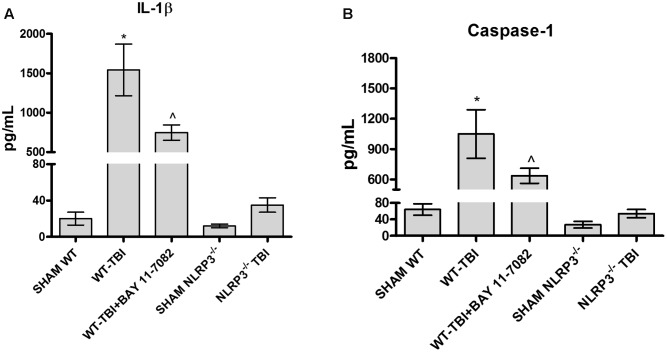
The graph **(A)** represents the levels of IL-1b in brain lysates from each group of animals 24 h after TBI. The graph **(B)** represents the levels of caspase-1 in brain lysates from each group of animals 24 h after TBI. ^∗^*p* < 0.05 vs Sham WT; ˆ*p* < 0.05 vs WT-TBI. Each bar represents the mean and SD of seven animals.

IL-1b (**Figure 2A**) and caspase-1 (**Figure 2B**) levels were increased 24 h following TBI in brain lysates of WT mice, compared to Sham-WT (*p* < 0.0001). BAY 11-7082 administration in WT-TBI mice caused a marked decrease of both IL-1b and caspase-1 (*p* < 0.001), thus confirming its role as NLRP3 inhibitor. NLRP3^-/-^ mice showed almost undetectable levels of both IL-1b and caspase-1 and a not significant increase was observed following TBI (**Figure 2A**, *F* = 137.5; and **Figure 2B**, *F* = 116.2). These data indicate that NLRP3 is activated during TBI in WT mice, whereas the lack of NLRP3 in TBI-NLRP3^-/-^ mice and NLRP3 blockade is protective, reducing in turn inflammation and ameliorating the prognosis of the injury.

### Lack and Blockade of NLRP3 Preserve Histological Features

**FIGURE 3:**
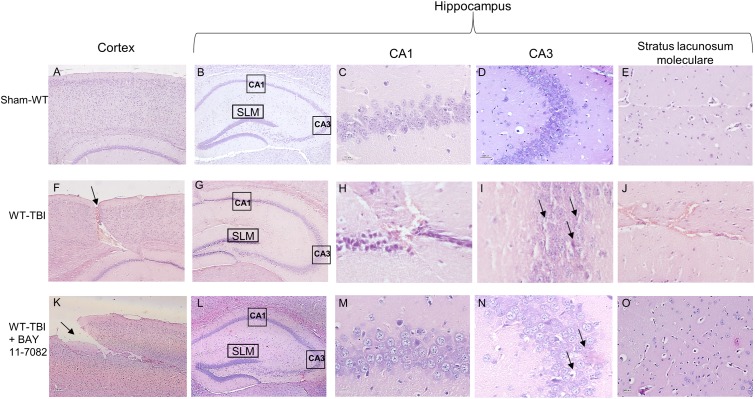
Representative H&E staining of brain tissue from WT animals 24 h following TBI. **(A)** cortex of Sham-WT animal. Original magnification X5. **(B)** hippocampus of Sham-WT animal. The rectangle indicates the Ca1, Ca3, and stratus lacunosum moleculare areas used for enlargement. Original magnification X5. **(C)** Ca1 area of Sham-WT animal. Original magnification X40. **(D)** Ca3 area of Sham-WT animal. Original magnification X40. **(E)** Stratus lacunosum moleculare of Sham-WT animal. Original magnification X20. **(F)** cortex of WT-TBI animal, the arrow indicates the impact point, hemorrhage and edema are markedly visible. Original magnification X5. **(G)** hippocampus of WT-TBI animal. The rectangle indicates the Ca1 and Ca3 areas used for enlargement. Original magnification X5. **(H)** Ca1 area of WT-TBI animal, showing loss of normal architecture, presence of hemorrhage and edema, with shrank neurons. Original magnification X40. **(I)** Ca3 area of WT-TBI animal, showing diffused neuronal loss and presence of eosinophil neurons (arrows). Original magnification X40. **(J)** Stratus lacunosum moleculare of WT-TBI animal, showing hemorrhage and edema. Original magnification X20. **(K)** Cortex of WT-TBI+BAY 11-7082 animal, the arrow indicates the impact point, hemorrhage and edema are markedly visible. Original magnification X5. **(L)** Hippocampus of WT-TBI+BAY 11-7082 animal. The rectangle indicates the Ca1, Ca3, and stratus lacunosum moleculare areas used for enlargement. Original magnification X5. **(M)** Ca1 area of WT-TBI+BAY 11-7082 animal, showing a more preserved architecture, without hemorrhage or edema. Original magnification X40. **(N)** Ca3 area of WT-TBI+BAY 11-7082 animal, showing slight neuronal loss and presence of shrank and eosinophil neurons (arrows). Original magnification X40. **(O)** Stratus lacunosum moleculare of WT-TBI+BAY 11-7082 animal, showing a more preserved architecture. Original magnification X20.

**FIGURE 4:**
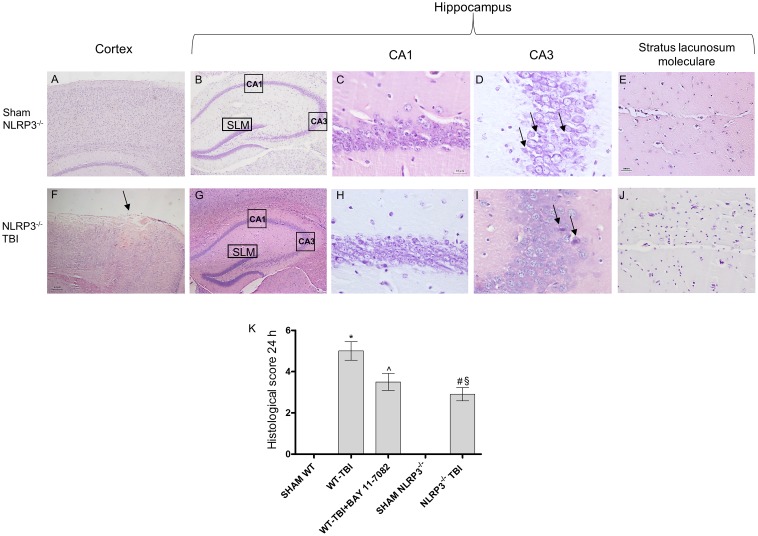
Representative H&E staining of brain tissue from NLRP3^-/-^ mice 24 h following TBI. **(A)** Cortex of Sham NLRP3^-/-^ animal. Original magnification X5. **(B)** Hippocampus of Sham NLRP3^-/-^ animal. The rectangle indicates the Ca1, Ca3, and stratus lacunosum moleculare areas used for enlargement. Original magnification X5. **(C)** Ca1 area of Sham NLRP3^-/-^ animal. Original magnification X40. **(D)** Ca3 area of Sham NLRP3^-/-^ animal. Original magnification X40. **(E)** Stratus lacunosum moleculare of Sham NLRP3^-/-^ animal. Original magnification X20. **(F)** Cortex of NLRP3^-/-^-TBI animal, the arrow indicates the impact point, hemorrhage and edema are markedly visible. Original magnification X5. **(G)** Hippocampus of NLRP3^-/-^-TBI animal. The rectangle indicates the Ca1, Ca3, and stratus lacunosum moleculare areas used for enlargement. Original magnification X5. **(H)** Ca1 area of NLRP3^-/-^-TBI animal, showing a preserved architecture, absence of hemorrhage and edema, with few shrank neurons. Original magnification X40. **(I)** Ca3 area of NLRP3^-/-^-TBI animal, showing neuronal loss and presence of eosinophil neurons (arrows). Original magnification X40. **(J)** Stratus lacunosum moleculare of NLRP3^-/-^-TBI animal, showing an almost normal architecture. Original magnification X20. **(K)** The graph represents the cumulative histological score evaluated at 24 hours from each group of animals. ^∗^*p* < 0.05 vs Sham WT; ˆ*p* < 0.05 vs WT-TBI; #*p* < 0.05 vs Sham NLRP3^-/-^; ^§^ *p* < 0.05 vs WT-TBI. Each bar represents the mean and SD of seven animals.

At 24 h post-TBI the damage was evident in the cortex of both WT (*p* < 0.0001) and NLRP3^-/-^ (*p* < 0.0005) mice, compared to sham animals (**Figures 3**, **4**). TBI-WT mice showed histological alterations with scattered axonal injury, perilesional infarcted areas, disorganized tissue a strong inflammatory infiltrate and oedema (**Figures 3**, **4**). At hippocampal level neuronal loss was evident in the CA1 and CA3 with neuronal shrinking and eosinophilia (**Figures 3**, **4**). The use of BAY 11-7082 as NLRP3 blocking agent resulted in efficient reduction of inflammatory infiltrate and damage at both cortex and hippocampus level (**Figures 3**, **4K**), as compared to TBI-WT animals (*p* < 0.01).

TBI-NLRP3^-/-^ animals had decreased (*p* < 0.01) inflammatory infiltrate and oedema compared to TBI-WT mice (**Figures 3**, **4**) with more preserved neurons, especially in the hippocampus (**Figures 3**, **4**), suggesting that mice lacking inflammasome might be protected during TBI, at least in the first 24 h.

**FIGURE 5:**
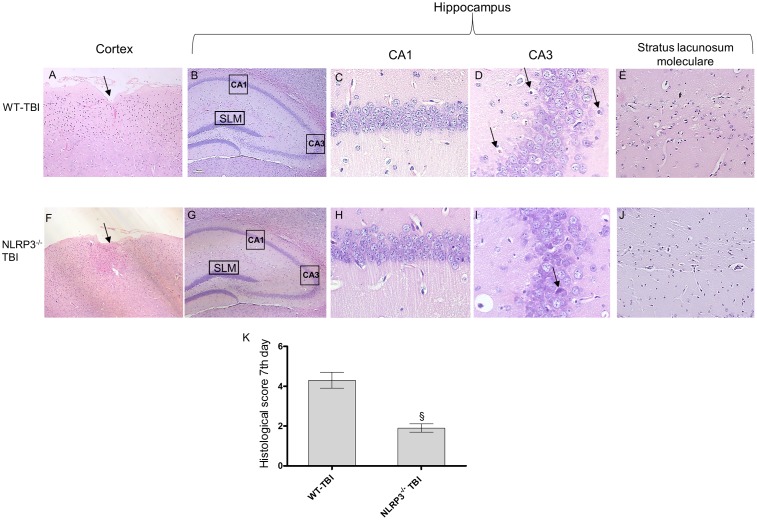
Representative H&E staining of brain tissue from WT and NLRP3^-/-^ mice 7 days following TBI. **(A)** Cortex of WT-TBI animal, the arrow indicates the impact point, hemorrhage and edema are still markedly visible. Original magnification X5. **(B)** hippocampus of WT-TBI animal. The rectangle indicates the Ca1, Ca3, and stratus lacunosum moleculare areas used for enlargement. Original magnification X5. **(C)** Ca1 area of WT-TBI animal, showing partial restoration of normal architecture with shrank neurons. Original magnification X40. **(D)** Ca3 area of WT-TBI animal, showing neuronal loss and presence of eosinophil neurons (arrows). Original magnification X40. **(E)** Stratus lacunosum moleculare of WT-TBI animal, showing partially restored architecture. Original magnification X20. **(F)** Cortex of NLRP3^-/-^-TBI animal, the arrow indicates the impact point, hemorrhage and edema are markedly visible. Original magnification X5. **(G)** Hippocampus of NLRP3^-/-^-TBI animal. The rectangle indicates the Ca1, Ca3, and stratus lacunosum moleculare areas used for enlargement. Original magnification X5. **(H)** Ca1 area of NLRP3^-/-^-TBI animal, showing a preserved architecture, absence of hemorrhage and edema, with few shrank neurons. Original magnification X40. **(I)** Ca3 area of NLRP3^-/-^-TBI animal, showing neuronal loss and presence of eosinophil neurons (arrows). Original magnification X40. **(J)** Stratus lacunosum moleculare of NLRP3^-/-^-TBI animal, showing an almost normal architecture. Original magnification X20. **(K)** The graph represents the cumulative histological score evaluated at 7 days from each group of animals. §*p* < 0.05 vs WT-TBI. Each bar represents the mean and SD of seven animals.

At day 7 post-injury the impact point was still evident in both strain of mice; WT animals showed an altered structure of nerve fibers and diffused oedema (**Figure 5**). In the hippocampal areas shrunken neurons and vacuolar degeneration were suggestive of extensive damage (**Figure 5**). In TBI-NLRP3^-/-^ mice the cortex architecture was more conserved with a strong decrease (*p* < 0.01) in both oedema and inflammation in the perilesional area. In the hippocampus the reduced eosinophilia and the almost complete absence of shrunken neurons encourages the hypothesis that NLRP3 inflammasome absence might be protective for the relatively long-term sequelae of TBI.

### NLRP3 Absence Reduces BAX Expression

**FIGURE 6:**
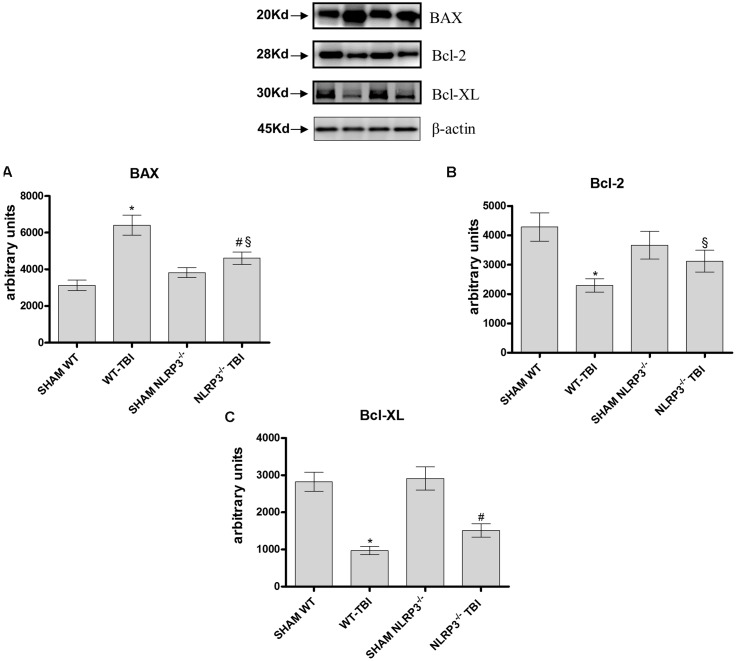
Representative western blot of Bax, Bcl-2 and Bcl-xl at dat 7. The graphs represent the quantification of Bax **(A)**, Bcl-2 **(B)**, and Bcl-xl **(C)**. ^∗^*p* < 0.05 vs Sham WT; ^#^*p* < 0.05 vs Sham NLRP3^-/-^; ^§^*p* < 0.05 vs WT-TBI. Each bar represents the mean and SD of seven animals.

The pro-apoptotic BAX protein was significantly expressed in hippocampal lysate from WT-TBI mice compared to Sham WT animals (*p* < 0.0001). NLRP3^-/-^ mice subjected to TBI demonstrated a reduced activation of BAX compared to WT-TBI (*p* < 0.01), suggesting that the lack of the inflammasome lead to a blunted apoptotic activation (**Figure 6A**, *F* = 187.32). On the other hand, the anti-apoptotic proteins Bcl-2 and BCL-XL were markedly reduced in both WT (*p* < 0.0001) and NLRP3^-/-^ (*p* < 0.005) animals subjected to TBI as compared to the relative Sham mice (**Figure 6B**, *F* = 30.94; **Figure 6C**, *F* = 22.35).

## Discussion

The few papers, so far published on the role of NLRP3 in TBI used indirect strategies to prevent NLRP3 activation (Liu et al., 2013; Dong et al., 2016; Ma et al., 2016; Wei et al., 2016; Lin et al., 2017). A study using telmisartan in mice subjected to cold brain injury showed that the treatment reduced oxidative stress and brain oedema, as a consequence NLRP3 and IL-1b production were decreased; however, the effect on this pathway is rather indirect than direct (Wei et al., 2016).

Similarly, another paper studying the effect of propofol in rats subjected to blast TBI demonstrated a reduced NLRP3 activation and a consequent ameliorated brain damage due to the antioxidant and ROS scavenger effect of propofol (Ma et al., 2016). This NLRP3 blocking strategy is again rather indirect than direct. Finally, in a mouse model of subarachnoid hemorrhage melatonin blunted NLRP3 activation reducing the levels of ROS and other oxidants, improving neuron survival (Dong et al., 2016).

So far, the present study is the first report about the use of a direct NLRP3 blocking agent (BAY 11-7082) and NLRP3^-/-^ mice. TBI resulted in impaired cognitive processes such as attention and memory, this altered brain function has been related to the loss of several molecules as EGR1, which plays a role in memory retention and has been shown reduced following TBI (Michael et al., 2005). Our results show that NLRP3^-/-^ and WT animals treated with BAY 11-7082 had an ameliorated memory performance in the first 24 h following TBI, with an increased EGR1 protein expression compared to TBI-WT animals. In fact, both in NLRP3^-/-^ and WT mice treated with BAY 11-7082 the histology revealed a more conserved brain tissue, with reduced oedema, inflammatory infiltrate and apoptosis. Brain oedema reflects a disruption of the blood brain barrier, which is primarily induced by endothelial cells apoptosis, degradation of the basal-lamina by proteases, and diffuse inflammatory reaction that further contributes to NLRP3 activation. We used a dual experimental strategy to investigate the role of NLRP inflammasome in TBI: NLRP3 knock out mice and a direct pharmacological blockade of the inflammasome. In our model, the lack of NLRP3 as well as the administration of BAY 11-7082, determined a strong reduction in IL-1β and Caspase-1 levels that are further responsible for amplifying brain damage following TBI. Despite neurons may produce IL-1b through the NLRP1 and not NLRP3 activation pathway (Kaushal et al., 2015), and NLRP3 inflammasome is particularly expressed in microglia, it is reasonable to speculate that the improved histological picture and reduced inflammatory mediators observed in our experimental setting are due to a reduced glial activation. In fact, microglial activation is one of the main process involved in the propagation of inflammation, which may lead to impaired neuronal survival as a consequence of the production of cytokines and ROS, also during TBI (d’Avila et al., 2012).

Several factors contribute to the characteristic alterations of TBI, especially the hyper production of damage-associated molecular patterns (DAMPS), ROS, and ATP accumulation; in fact all these insults can activate the NLRP3 inflammasome (Schroder and Tschopp, 2010). However, the increased production of ROS is also involved in the induction of cell death mechanisms (Szabo and Dawson, 1998). BAX is a pro-apoptotic protein and its activation is responsible for developmental cell death (Chittenden et al., 1995) also during brain injuries (Bar-Peled et al., 1999). On the contrary, the Bcl-2 family is the most expressed anti-apoptotic group of proteins in the central nervous system. Bcl-2 and Bcl-XL overexpression has a significant neuroprotective function in the mature nervous system, in addition, the administration of BCL-XL protein during brain ischemia increased neuronal survival (Cao et al., 2002). Furthermore, it has been demonstrated that the silencing of NLRP3 or ASC in cells decreased pro-apoptotic proteins and increased anti-apoptotic ones (Shen et al., 2016).

Although inflammasome activation is associated with pyroptosis by caspase 1 and IL-1b release, also apoptotic process may be activated by ASC through caspase 8 recruitment (Grabinger et al., 2013; Wallach et al., 2016).

In agreement with these previous observations NLRP3^-/-^ mice subjected to TBI showed a significant reduction in the pro-apoptotic protein BAX and a preserved Bcl-2 and BCL-XL protein expression, indicating that despite the injury determined neural disruption and loss of function, the absence of the NLRP3 is a protective factor. Finally, the evidence showing that BAY 11-7082 protects mice from TBI, corroborates this hypothesis.

Collectively, these data show that the NLRP3 inflammasome might be a therapeutic target following TBI, to modulate both inflammation and apoptosis and possibly preventing the long-term sequeale. However, further experiments will be needed to better explore the therapeutic potential of inhibiting the NLRP3 pathway.

## Author Contributions

NI and AB conceived and designed the study; MC, GvP, GrP, FF, FM, AD, VF, CF, and LM performed the data; NI, AB, EM, PB, and VA analyzed and interpreted data. FS and DA drafted and critically revised the paper.

## Conflict of Interest Statement

The authors declare that the research was conducted in the absence of any commercial or financial relationships that could be construed as a potential conflict of interest.
