# Correction: Lack of the Nlrp3 inflammasome improves mice recovery following traumatic brain injury

**DOI:** 10.3389/fphar.2025.1733683

**Published:** 2025-11-26

**Authors:** Natasha Irrera, Gabriele Pizzino, Margherita Calò, Giovanni Pallio, Federica Mannino, Fausto Famà, Vincenzo Arcoraci, Vincenzo Fodale, Antonio David, Francesca Cosentino, Letteria Minutoli, Emanuela Mazzon, Placido Bramanti, Francesco Squadrito, Domenica Altavilla, Alessandra Bitto

**Affiliations:** 1 Department of Clinical and Experimental Medicine, AOU Policlinico G. Martino, University of Messina, Messina, Italy; 2 Department of Veterinary Sciences, University of Messina, Messina, Italy; 3 Department of Human Pathology, AOU Policlinico G. Martino, University of Messina, Messina, Italy; 4 IRCCS Centro Neurolesi “Bonino-Pulejo”, Messina, Italy; 5 Department of Biomedical and Dental Sciences and Morphological and Functional Sciences, AOU Policlinico G. Martino, University of Messina, Messina, Italy

**Keywords:** NLRP3 inflammasome, traumatic brain injury, inflammation, cytokines, apoptosis

In the published article, there was an error in Figures 3B,D, 5B,D,G,I as published. The previously published images were erroneously labeled in the source file**.** The corrected Figures 3B,D, 5B,D,G,I and their captions appear below.

**FIGURE 3:**
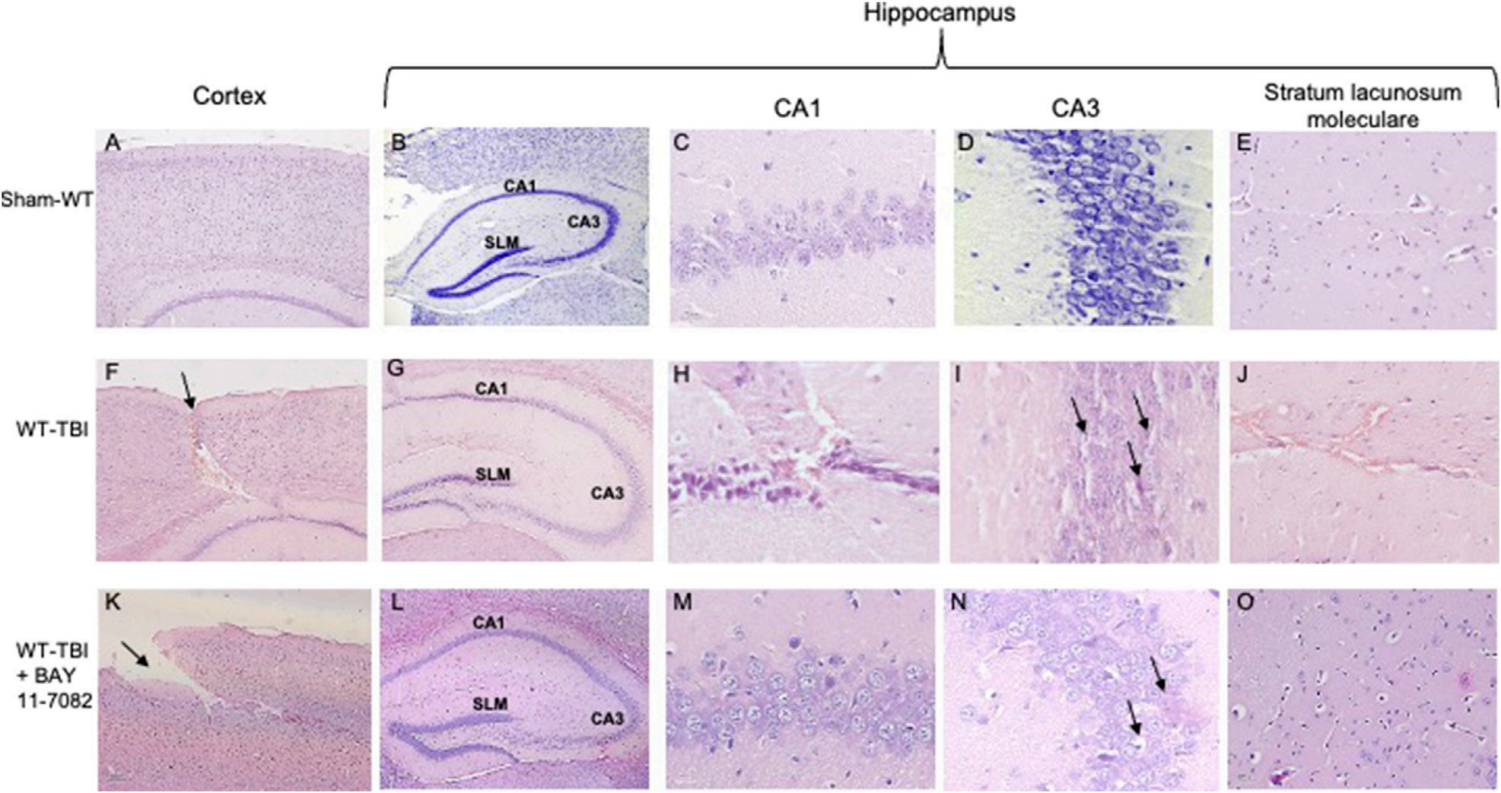
Representative H&E staining of brain tissue from WT animals 24 h following TBI. **(A)** Cortex of Sham-WT animal. Original magnification X5. **(B)** Hippocampus of Sham-WT animal. The rectangle indicates the Ca1, Ca3, and stratus lacunosum moleculare areas used for enlargement. Original magnification X5. **(C)** Ca1 area of Sham-WT animal. Original magnification X40. **(D)** Ca3 area of Sham-WT animal. Original magnification X40. **(E)** Stratus lacunosum moleculare of Sham-WT animal. Original magnification X20. **(F)** Cortex of WT-TBI animal, the arrow indicates the impact point, hemorrhage and edema are markedly visible. Original magnification X5. **(G)** Hippocampus of WT-TBI animal. The rectangle indicates the Ca1 and Ca3 areas used for enlargement. Original magnification X5. **(H)** Ca1 area of WT-TBI animal, showing loss of normal architecture, presence of hemorrhage and edema, with shrank neurons. Original magnification X40. **(I)** Ca3 area of WT-TBI animal, showing diffused neuronal loss and presence of eosinophil neurons (arrows). Original magnification X40. **(J)** Stratus lacunosum moleculare of WT-TBI animal, showing hemorrhage and edema. Original magnification X20. **(K)** Cortex of WT-TBI + BAY 11-7082 animal, the arrow indicates the impact point, hemorrhage and edema are markedly visible. Original magnification X5. **(L)** Hippocampus of WT-TBI + BAY 11-7082 animal. The rectangle indicates the Ca1, Ca3, and stratus lacunosum moleculare areas used for enlargement. Original magnification X5. **(M)** Ca1 area of WT-TBI + BAY 11-7082 animal, showing a more preserved architecture, without hemorrhage or edema. Original magnification X40. **(N)** Ca3 area of WT-TBI + BAY 11-7082 animal, showing slight neuronal loss and presence of shrank and eosinophil neurons (arrows). Original magnification X40. **(O)** Stratus lacunosum moleculare of WT-TBI + BAY 11-7082 animal, showing a more preserved architecture. Original magnification X20.

**FIGURE 5:**
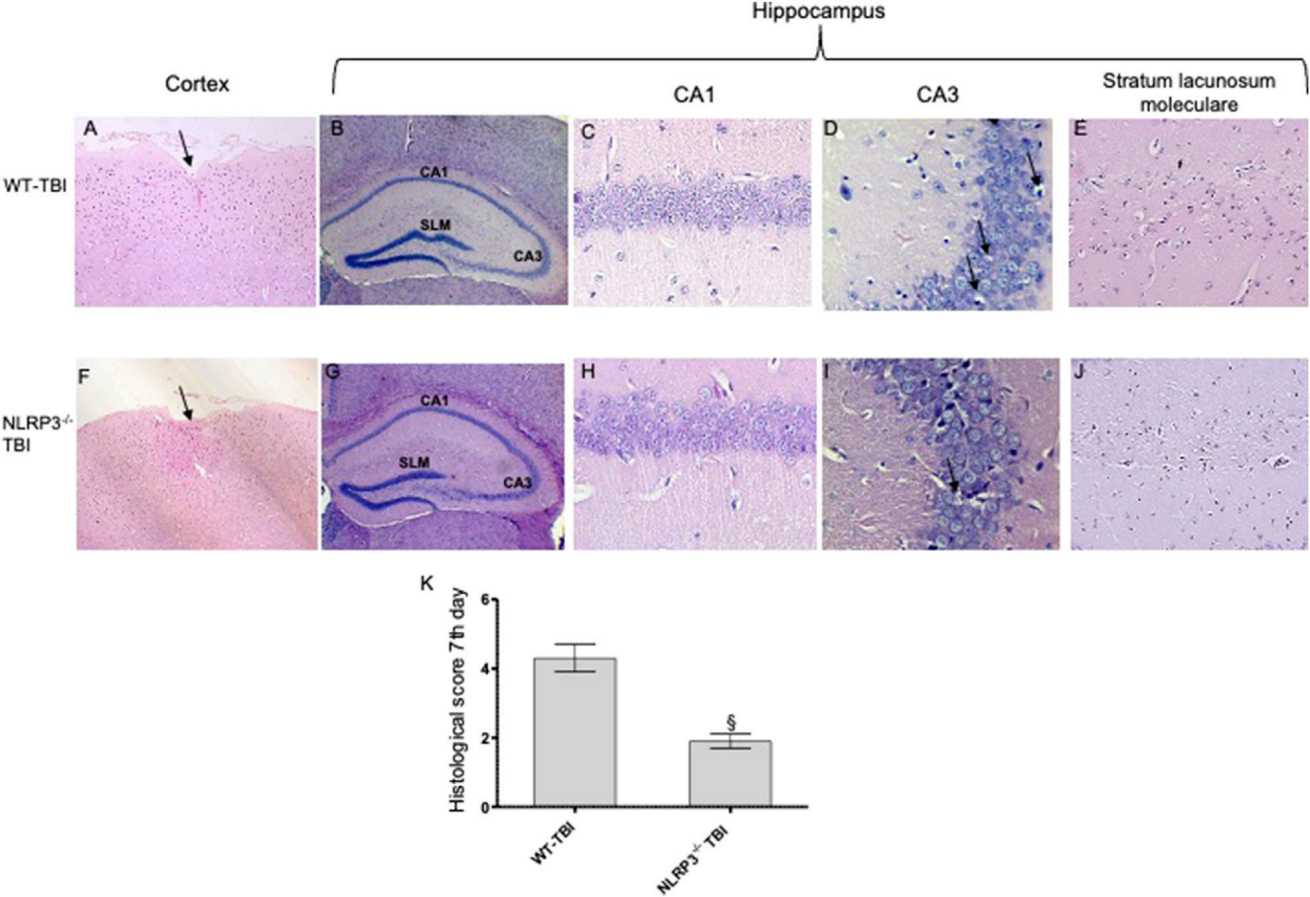
Representative H&E staining of brain tissue from WT and NLRP3^−/−^ mice 7 days following TBI. **(A)** Cortex of WT-TBI animal, the arrow indicates the impact point, hemorrhage and edema are still markedly visible. Original magnification X5. **(B)** Hippocampus of WT-TBI animal. The rectangle indicates the Ca1, Ca3, and stratus lacunosum moleculare areas used for enlargement. Original magnification X5. **(C)** Ca1 area of WT-TBI animal, showing partial restoration of normal architecture with shrank neurons. Original magnification X40. **(D)** Ca3 area of WT-TBI animal, showing neuronal loss and presence of eosinophil neurons (arrows). Original magnification X40. **(E)** Stratus lacunosum moleculare of WT-TBI animal, showing partially restored architecture. Original magnification X20. **(F)** Cortex of NLRP3−/−-TBI animal, the arrow indicates the impact point, hemorrhage and edema are markedly visible. Original magnification X5. **(G)** Hippocampus of NLRP3−/−-TBI animal. The rectangle indicates the Ca1, Ca3, and stratus lacunosum moleculare areas used for enlargement. Original magnification X5. **(H)** Ca1 area of NLRP3−/−-TBI animal, showing a preserved architecture, absence of hemorrhage and edema, with few shrank neurons. Original magnification X40. **(I)** Ca3 area of NLRP3−/−-TBI animal, showing neuronal loss and presence of eosinophil neurons (arrows). Original magnification X40. **(J)** Stratus lacunosum moleculare of NLRP3−/−-TBI animal, showing an almost normal architecture. Original magnification X20. **(K)** The graph represents the cumulative histological score evaluated at 7 days from each group of animals. §p < 0.05 vs. WT-TBI. Each bar represents the mean and SD of seven animals.

Author Francesca Cosentino was incorrectly written as Cosentino Francesca.

The original article has been updated.

